# On the Difficulty of Always Making an Exact Diagnosis in Typhoid Fever

**Published:** 1862-11

**Authors:** G. C. Paoli

**Affiliations:** Chicago


### ON THE DIFFICULTY OF ALWAYS MAKING AN EXACT DIAGNOSIS IN TYPHOID FEVER.

By G. C. PAOLI, M. D., Chicago.

I frankly admit that sometimes it is difficult for us to make a clear diagnosis in Typhoid Fever, though there may be many among us who doubt it, particularly those who have not an opportunity to make a post mortem examination. But when we consider how manifold are the symptoms of this disease, that the skin as well as the inner organs can be affected, and that when these organs are affected in their tissues, other symptoms arise, which are characteristic of another disease; while they are more constant in Typhoid Fever, we see them sometimes less predominant and sometimes wanting. And when we take in consideration the violence with which the disease sometimes appears, it is easily seen that the general symptoms sometimes bewilder us. It is also in this disease that we often find the greatest disproportions between the disease and the pathological changes. Thus we find the most violent cerebral symptoms can be present without any particular pathological change.

As a proof that Typhoid Fever can sometimes be compounded with Phthisis, a single case which I had under my treatment in Chicago will sufficiently illustrate. I will first relate the case, and then endeavor to place the symptoms separate from each other, so as to clearly present the difficulties of the diagnosis.

Louis Brenner, 28 years of age, of phthisical extraction, had suffered in childhood from scrofula, and had occasionally expectorated blood. Four weeks previous to the time I first saw him, he was attacked by fever, with frequent cough, but without any expectoration or dyspnœa. Pain behind os sternum; bowels irregular, rather loose; thirst and no appetite; had been very debilitated; had perspired a great deal during the night, and felt chilly during the day.

The 1st of August, when I saw him, he had had a very violent cough, and sometimes vomiting during the attack; no expectoration; pulse 106; respiration 20; skin moist and warm; a general debility; no appetite, very thirsty, tongue clean and aphthous. His bowels had had no evacuation in two days. He was very emaciated.

Percussion the left, regio infraclavicularis little dull; auscultation presented nothing else than spread mucous rales. I prescribed Quinine and Expectorants.

August 2d—He slept well; the cough somewhat milder, no expectoration; the tongue as the previous day; the pulse 96; auscultation; the right regio infraclavicularis somewhat dull.

August 3d—The cough very frequent and difficult; the expectoration mucous and tenacious; pulse 110; diarrhœa.

August 4th—The cough and the expectoration the same; the diarrhœa has increased. I prescribed Dover's Powder, continued with the Quinine. The patient complained of sweating during the whole night.

August 5th—Chill and profuse sweat during the whole night. Pulse 104; abdomen painful to the touch; diarrhœa somewhat less.

August 6th—No sleep. The patient deaf; the tongue red and clean; pulse 120; diarrhœa; the skin warm and dry.

August 7th—No sleep; the deafness rather increased; the expectoration purulent; tongue clean and aphthous; very thirsty and continued night sweat.

August 8th—No sleep; delirium. The patient remained deaf. The expectoration purulent; abdomen distended and tender to the touch; pulse 130; debility increased; auscultation gave no sign of phthisis.

August 9th—Heaviness in the head, accompanied with dizziness; shaking motion of the hands; diarrhœa the same; expectorations thick and purulent.

August 10th—No sleep; the pulse 128; tongue somewhat covered. On os sacrum I observed echymose. The patient perspired during the night.

11th—In the morning he died.

*Post Mortem Examination.*—The brain of healthy appearance.

The Lungs.—In the back part of the lower lobes I observed a hypostastic blood infiltration; in the right lung I observed small tubercules which had not the tubercular consistency, but were rather deposits of a peculiar nature. Pericardium natural; the heart somewhat relaxed, otherwise natural; the liver of natural size and consistency; the spleen somewhat larger than ordinary, also more solid and somewhat darker than in the natural state; the mesenteric glands neither hypertrophied, nor infiltration of blood; the stomach, duodenum and the greater part of jejunum healthy. In the lower part of jejunum the typhoid patches began to appear, which were more plainly visible in ileum, near to cœcum, where an extended ulceration appeared, and where the transverse muscular fibres were laid open, and in some places these fibres had disappeared and only the peritoneal membrane was visible.

On reading through these historia morbus, some perhaps might remark, how could such a case be considered phthisis, when not a few symptoms of typhoid were present?

I do not deny that this case was not with certainty considered Phthisis, because my treatment was that of Typhoid Fever; but, under the same treatment, could not the question arise whether the disease could not as well be considered as an acute Phthisis as Typhoid Fever, and such question I shall in the following try to sift and answer.

To make the question as plain as possible without being tedious, I will go through the symptoms and try to show how the same could be considered for both diseases, or taken singly, more for one than for the other. The facts which spoke for Phthisis were the patient's slender build, (Habitus tenor,) phthisical dispositions, scrofulous symptoms in his childhood, expectorations of blood with cough for several years, and then for the last four weeks with fever connected, dry cough with dyspnœa. Later, purulent expectorations, with profuse sweating and great emaciation. What argued for the Typhoid Fever were the nervous symptoms and congestion of the brain, namely, deafness, delirium and heaviness in the head, shaking of the hands and the ecchymosis on os sacrum a short time before he died—symptoms which are present in both diseases, namely, the appearance of the tongue, diarrhœa, pain upon pressure on the abdomen, and the loss of strength. In regard to the auscultation and percussion, it is clear that they can not give any certain result, when the tubercles are somewhat equally distributed in both lungs, and if the individual is emaciated then will a clear percussion take place over the whole chest. Auscultation will not either, under these circumstances, give any abnormal sound, because the healthy tissues situated between the tubercles are sufficient to give a natural sound. The more or less mucous rales which, under these circumstances, can be more or less present, depend often on affections of the mucous membrane of bronchia, which often follows upon tuberculous depositions of the lungs.

The pathological state in acute Phthisis is characterized by equal distributions of crude tubercles in both lungs, which sometimes are soft, but scarcely glue together and produce caverns. It is therefore clear that we cannot depend on percussion as a diagnostical sign. I will only mention that there can be no question about chronic Phthisis where caverns and large tuberculous agglomerations give us unmistakable signs.

I will now mention some of the symptoms which are peculiar to both diseases, so as to arrive at a conclusion how it was possible in these precisely to diagnose it Typhoid Fever.

I will commence with the symptoms which depend on a sickly state of the mucous membrane of the lungs :

1st. *Cough.*—This had been very severe, particularly during the night, sometimes to such a degree that it produced vomiting. This cough was very much like that which accompanies acute Phthisis, and, like it, characterised by its violence. When this accompanies Typhoid Fever the cough is generally very mild.

2. *Expectoration.*—In the commencement this was not present, but later it was first mucous and tenacious, and during the later period purulent. It therefore bore more resemblance to expectoration in Phthisis than Typhoid Fever, where it is more mucous.

3. *Dyspnea.*—This we know is not very severe in Phthisis when the fever is not very high. When I saw the patient the respiration was 20; later 26. The light dyspnœa would rather strengthen the diagnosis for a Phthisis than for a Typhoid Fever.

*Signs of an Affection of the Intestines which might occur in both Diseases:*

1. *The Clean Tongue.*—This state of the tongue occurs more frequently in Phthisis than in Typhoid Fever, when the tongue is more dry and furred, but when the incrustation loosens it becomes clean, particularly when the disease is of long duration. When I saw the patient the tongue was aphthous, which frequently occurs in Phthisis, but when the nervous symptoms appeared it become dry and furred, which is characteristic of Typhoid Fever.

2. That these symptoms were not conclusive, but spoke more in favor of Phthisis than Typhoid Fever.

Diarrhœa is a symptom of ulceration in both diseases, so that this symptom can not determine the diagnosis, and furthermore, diarrhœa can sometimes cease for a time in both diseases and appear again. We could argue that the diarrhœa in Phthisis generally first appears when the disease is further advanced, but we know that sometimes the deposition of tubercles commences earlier in the intestines than in the lungs, and that a diarrhœa can prove fatal to the patient before auscultations and percussions can diagnose the presence of tubercles of the lungs.

3. Pain which the patient felt by pressure on the abdomen is a symptom in both diseases. The Phthisical ulcerations, as well as the Typhoid, have mostly their site in the region of the cœcum.

4. Emaciation is well known to be equally great in Typhoid Fever as in Phthisis, because the diarrhœa in both diseases is the cause of the emaciation. Experience has taught us that a patient whose lungs are filled up with tubercles does not emaciate in such a degree as when diarrhœa is present.

5. The nervous symptoms which appeared in the latter stage of the disease is the only sign which strengthened our diagnosis of a Typhoid Fever; still even this could be present in Phthisis, but this is very seldom the case.

6. The sweat, which we know is a constant symptom in Phthisis, but we rarely observe that it continues steadily in Typhoid Fever.

